# Pollen Alters Amino Acid Levels in the Honey Bee Brain and This Relationship Changes With Age and Parasitic Stress

**DOI:** 10.3389/fnins.2020.00231

**Published:** 2020-03-24

**Authors:** Stephanie L. Gage, Samantha Calle, Natalia Jacobson, Mark Carroll, Gloria DeGrandi-Hoffman

**Affiliations:** Carl Hayden Bee Research Center, Agricultural Research Service, United States Department of Agriculture, Tucson, AZ, United States

**Keywords:** *Nosema ceranae*, insect, learning, memory, proboscis extension reflex, nutrition, nurse bees

## Abstract

Pollen nutrition is necessary for proper growth and development of adult honey bees. Yet, it is unclear how pollen affects the honey bee brain and behavior. We investigated whether pollen affects amino acids in the brains of caged, nurse-aged bees, and what the behavioral consequences might be. We also tested whether parasitic stress altered this relationship by analyzing bees infected with prevalent stressor, *Nosema ceranae*. Levels of 18 amino acids in individual honey bee brains were measured using Gas Chromatography – Mass Spectrometry at two different ages (Day 7 and Day 11). We then employed the proboscis extension reflex to test odor learning and memory. We found that the honey bee brain was highly responsive to pollen. Many amino acids in the brain were elevated and were present at higher concentration with age. The majority of these amino acids were non-essential. Without pollen, levels of amino acids remained consistent, or declined. *Nosema*-infected bees showed a different profile. Infection altered amino acid levels in a pollen-dependent manner. The majority of amino acids were lower when pollen was given, but higher when pollen was deprived. Odor learning and memory was not affected by feeding pollen to uninfected bees; but pollen did improve performance in *Nosema*-infected bees. These results suggest that pollen in early adulthood continues to shape amino acid levels in the brain with age, which may affect neural circuitry and behavior over time. Parasitic stress by *N. ceranae* modifies this relationship revealing an interaction between infection, pollen nutrition, and behavior.

## Introduction

The honey bee brain has evolved a remarkable complexity. The bee brain is a small and compact structure that contains approximately 960,000 neurons and is similar in size to a sesame seed (Giurfa, 2003). Despite the small size, honey bees exhibit sophisticated capabilities akin to higher-order cognition (Giurfa, 2003). Bees see the world in color, distinguish a range of odors, perceive shapes and patterns, and deftly navigate their terrain (Srinivasan, 2010). These capabilities likely evolved for honey bees to survive in dynamic environments in which food sources ebb and flow. Honey bees must rely on complex sensory and motor systems to forage for pollen and nectar, communicate the location and value of these resources, distinguish between hive odors to nurse developing larvae, defend their colonies from intruders; and perform many other nuanced behaviors to ensure colony survival (Pahl et al., 2010).

To fuel these activities, adequate nutrition is necessary for growth and development (De Groot, 1953). Pollen is the primary source for amino acids and lipids, while nectar and honeydew are the natural carbohydrate sources. The consumption of these resources depends upon the age of the bee. Pollen is consumed primarily during the first 3 to 5 days of an adult worker’s life (Hagedorn, 1967). Nectar, on the other hand, is transformed into honey inside the hive and is consumed throughout the bee’s lifespan (De Groot, 1953; Haydak, 1970). Though a honey bee is considered an adult after emergence, there is substantial growth that occurs during the first 6 days (De Groot, 1953). There is an increase in body weight and total protein content increases by 25–50% (Haydak, 1934). To support this growth, honey bees consume considerable amounts of pollen, which may begin as early as 1–2 h post-emergence; reaching a maximum by Day 5 (Hagedorn, 1967). The hypopharyngeal glands, fat body, and other internal organs develop simultaneously. If pollen is not available, growth is stunted. Bees experience a loss of weight, diminished protein content, and a reduced lifespan (Haydak, 1970).

As honey bees age, they perform a series of age-dependent behaviors or polyethism. The youngest adults perform tasks such as comb cleaning, nursing developing larvae or tending the queen. Bees will then switch to “out of hive” tasks such as guarding and foraging. The honey bee brain shows predictable patterns of change related to transitions in behavior (Withers et al., 1993; Mercer, 2001). For instance, the mushroom body, which supports olfactory memory, navigation, and behavioral choice, increases in volume in foraging bees and precocious foragers (Fahrbach and Robinson, 1996; Fahrbach et al., 2003). In the antennal lobe, the primary neuropil for olfactory processing, individual glomeruli change in volume and number of synapses with the onset of foraging (Brown et al., 2004). Interestingly, the plastic nature of the adult honey bee brain is not due to neurogenesis (Fahrbach et al., 1995). Instead, it is presumed that structural plasticity is dependent upon changes to existing neurons and networks (Farris et al., 2001; Mercer, 2001). These networks are modified by neurotransmitters, neuromodulators, and neurohormones that elevate in concentration with the onset of foraging (Wagener-Hulme et al., 1999; Schulz et al., 2002a, b). Therefore, if pollen is necessary for growth and development of the bee, pollen may also be necessary for optimal brain development and behavior.

Amino acid levels in the brain may provide clues that connect pollen nutrition with neurodevelopment. All nutrients to an extent influence brain maturation, but protein, at least in humans and other mammals, appears most critical to the development of neurological functions (Morgane et al., 2002). Amino acids provide not only the building blocks of proteins and polypeptides, but play direct and indirect roles in neurotransmission. For example, amino acids can be precursors for neurotransmitters or be neurotransmitters themselves. They are also precursors of enzymes, neurohormones and neuropeptides. Less is known about how the honey bee brain uses amino acids from pollen. We know that the honey bee brain is plastic with age and undergoes structural and chemical changes, which involve amino acids. We also know that similar to other animals, the bee brain has metabolic needs that are distinct from the rest of the animal, and this metabolic capacity is higher in nurses than in foragers (Ament et al., 2008; Alaux et al., 2011). Yet, it is unclear whether amino acids from pollen affect the levels of amino acids in the brain, and whether levels change in the honey bee with age. Given that pollen is consumed in the first 5 days of adulthood, we hypothesized that pollen would have a lasting effect on amino acid levels with age and affect neural circuitry such that behavioral consequences could be measured.

This hypothesis was tested using nurse-aged bees at Day 7 and Day 11. Nutrition in nurses is well-studied, with known effects on nurse physiology and behavior (Crailsheim and Stolberg, 1989; Pernal and Currie, 2003; DeGrandi-Hoffman et al., 2010; Corby-Harris et al., 2014, 2016). In this experiment, pollen was made available to some nurses, and unavailable to others. We compared levels of individual amino acids in the whole brain of bees from both groups and tested olfactory learning and memory to see if there was a relationship between diet and cognition. This hypothesis was further expanded upon by examining bees with parasitic stress from *Nosema ceranae*. *N. ceranae* is a mid-gut parasite known to disrupt both energy metabolism (Higes et al., 2007; Holt et al., 2013; Vidau et al., 2014; Kurze et al., 2016) and olfactory-guided behavior (Gage et al., 2018). In testing infected bees, we sought to gain a better understanding of how stress, either nutritional or parasitic, affects the relationship between a pollen diet and brain function. With these parameters, we assessed how pollen influences amino acid composition in the brain, its impact on olfactory learning and memory, and how the brain may change with stress.

## Materials and Methods

### Animals

Brood frames were collected from colonies at the Carl Hayden Bee Research Center apiaries in Tucson, Arizona between October and November 2016. Frames, from three or more hives, were taken from European colonies, *Apis mellifera-L* headed by queens from Pendell Apiaries (Stoneyford, CA, United States). Newly emerged bees were obtained from sealed brood frames kept overnight in a temperature-controlled dark environmental room (32–34°C, 30–40% relative humidity). The emerged bees were separated into cages according to four treatment groups: (1) pollen-fed bees (+ P) (2) pollen-deprived bees (−P) (3) pollen-fed bees + *Nosema* (+ P + N) and (4) pollen-deprived bees + *Nosema* (−P + N). Bees were kept for 11 days in a Binder BD (E2) incubator (Binder GmbH, Tuttlingen, Germany) at 31.7°C with 40% relative humidity, under constant dark conditions.

### Feeding

**FIGURE 1:**
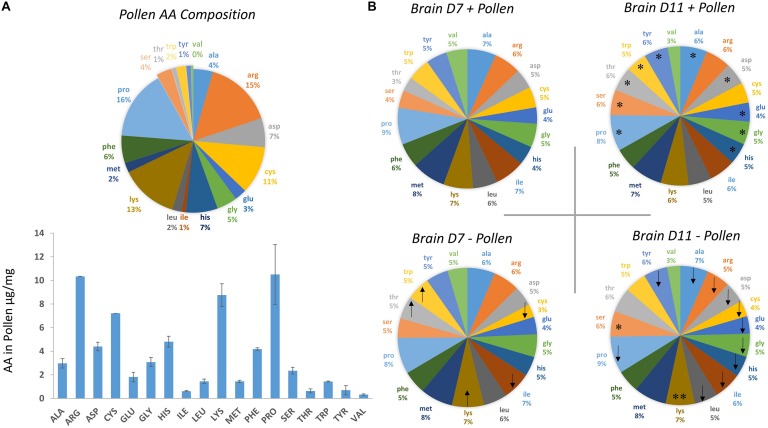
Amino acid (AA) composition of bee-collected pollen and whole bee brains ± pollen. **(A)** Amino acid composition of multi-floral, bee-collected pollen fed to bees. Pollen was collected during the fall season in Tucson, Arizona during 2016. **(B)** Amino acid composition of whole brain homogenates were measured from bees dissected on Day 7 and Day 11 post-eclosion. Bees were fed pollen (top, + pollen) and deprived of pollen (bottom, – pollen). Arrow direction denotes an increase (up) or decrease (down) in mean amino acid levels with pollen deprivation. For example, – pollen in Day 7 bees had lower average cysteine levels compared with + pollen Day 7 bees. Asterisks denote a significant change in amino acid levels from Day 7 to Day 11 within the same feeding group (Ex. Day 7 + pollen, Day 11 + pollen). ^∗^One asterisk denotes a significant increase in AA levels from Day 7 to Day 11, while ^∗∗^two asterisks denotes a decrease.

Bee-collected corbicular pollen was gathered from Tucson, Arizona during the months of September and early October 2016 and kept at 20°C until fed to bees. Each cage of 60 bees was given an insert with 3 g of ground pollen, a 50% sugar solution and water *ad libitum.* Sugar, water and pollen were changed at 7 days. Cages were checked daily and dead bees were removed. Fresh pollen was analyzed for amino acid content using Gas Chromatography – Mass Spectrometry (Figure 1). Identification of pollen species was determined via ITS sequencing as part of another study and included pollens *Pectis* sp. and *Corethrogyne* sp. from the daisy family, and *Ambrosia* sp. or ragweed.

### *Nosema inoculum*, Spore Counts, Species Detection

Spores to create the *Nosema* inoculum were collected from bees found at the entrance of an infected hive the day before, or the day of, the inoculation. Infected bee abdomens were crushed with a mortar and pestle in water and the *Nosema* inoculum was prepared using methods described in Fries et al. (2013). Individual bees were placed into an Eppendorf tube cut with a hole, which was large enough for a proboscis to extend through to feed. Bees in *Nosema* treatment groups (+N bees) were hand-fed a 2 μL inoculum containing 100,000 spores in a 50% cane sugar solution. Bees in control treatment groups were handled identically and fed 2 μL of 50% cane sugar solution. Bees were removed from individual tubes and placed into cages (60 bees per cage, two cages per treatment). Pollen, sugar and water were kept from cages for 1 h after feeding to prevent trophallaxis.

The effect of pollen on spore proliferation was determined using Day 7 bees from individuals used during the behavioral experiments. Twenty bees per treatment group were individually analyzed for spore intensity at Day 7 (Total = 80). Sample sizes were smaller on Day 11 as bees succumbed to the infection (*N* = 26). *Nosema* spore numbers per bee were determined by removing the abdomen, grinding it with a mortar and pestle in 1 mL of water, and placing a 10 μL sample on a hemocytometer (Fries et al., 2013). Five squares were counted, and spore number was estimated as described in Fries et al. (2013).

To determine the *Nosema* species used in this study, abdomens from two infected bees from the Day 11 + pollen group (16,583,333 and 6,150,000 spores counted) were used to extract *Nosema* DNA. The methods for the molecular detection of *Nosema* species, including PCR parameters, were detailed in Fries et al., 2013. In brief, abdomens were crushed in 1 mL of DI water, microcentrifuged and the supernatant was removed. The sample pellets were re-suspended in 100 μL. The samples were briefly bead-beat to rupture *Nosema* cell walls. The samples were combined, and DNA was extracted using the DNeasy Plant Mini Kit (Qiagen) beginning at Step #7 (lysis steps omitted, adequate with bead-beat) according to the manufacturer specifications. PCR was performed with 2X GoTaq master mix (Promega) and the following primers: *N. ceranae F (5′ – cggataaaagagtccgttacc – 3′)/N. ceranae R(5′ – tgagcagggttctagggat– 3′) and Nosema apis F (5′ –ccattgccggataagagagt– 3′/N. apis R (5′ – ccaccaaaaactcccaagag-3′)* (Invitrogen). Recombinant plasmid DNA containing *N. apis* and *N. ceranae* were generously provided by the Beltsville Laboratory (USDA-ARS) and served as positive controls. The PCR products were visualized using a 1% agarose gel stained with SYBR Safe (Invitrogen).

### Amino Acid Analysis of Brain Tissue and Pollen

Bees, on Day 7 and Day 11, were flash frozen in liquid nitrogen (*N* = 15 bees per treatment, *N* = 60 total). Bees were kept at −80°C until whole brains (including antennal and optic lobes) could be dissected. Each brain was rapidly dissected and weighed using a Sartorius CP2P microscale (Sartorius, Göttingen, Germany). Each brain was then frozen in liquid nitrogen and stored at −80°C until chemical analyses.

The details of the amino acid analyses on individual brains are described in Gage et al. (2018). In brief, conventional acid hydrolysis with chloroformate derivatization was used to quantify all amino acids, with the exceptions of tryptophan, cysteine, and arginine. Tryptophan was recovered using base hydrolysis with chloroformate derivatization, while cysteine and arginine were quantified with sodium azide acid hydrolysis followed by phenylisothiocyanate derivatization. Using conventional acid hydrolysis, asparagine and glutamine were hydrolyzed to their acidic forms (Fountoulakis and Lahm, 1998). As a result, asparagine and glutamine levels were measured as concentrations of aspartic acid and glutamic acid. Cysteine, too, was quantified in its oxidative form, cysteic acid. Conventional acid- and base hydrolyzed samples were analyzed using EZ: Faast Amino Acid Analysis Kit for Protein Hydrolyzates by Gas Chromatography – Mass Spectrometry (Phenomenex, Torrence, CA, United States). The re-dissolved chloroformate derivatives were analyzed by EI GC-MS on an Agilent 7890A gas chromatography system coupled with a 5975C EI mass spectrometer detector. Sodium azide hydrolyzed samples were analyzed using a modified method from Elkin and Wasynczuk (1987). The re-dissolved phenylthiocarbamyl derivatives were analyzed by reverse-phase HPLC-PDA on a Thermo Scientific Spectra System coupled with a Finnigan Surveyor PDA Plus Detector.

The methods to measure amino acids from pollen was published in DeGrandi-Hoffman et al. (2018). In brief, fresh pollen was ground, and six samples of 10 mg were homogenized using a mortar and pestle with liquid nitrogen. Each sample of 10 mg were sealed under nitrogen gas in a crimp vial and digested with 500 μL 6M HCl with 4% thioglycolic acid at 70°C for 24 h. To remove acid residues, 50 μL of the acid hydrosylate was filtered and dried in a Savant 2200 Speed Vac (Thermo Scientific, Inc.) The material was then re-solvated, derivatized, and separated by the EZ-FAAST kit protocol (Phenomenex). Amino acid composition in pollen (Figure 1) was determined using the hydrolysis and derivatization steps described above.

### Odor Learning and Memory

Learning and memory experiments took place in November and early December 2016. The night before associative-learning tests, sugar was removed from cages between 5 and 6 PM. The next morning, bees were restrained in a 1 mL pipette tip cut such that the body was restrained and the neck free to rotate. Wax was used around the opening for further restraint around the thorax. Bees were tested for proboscis extension reflex (PER) by applying a wooden applicator soaked in 50% cane sugar solution to the tip of the antennae. Bees were not allowed to lick. Only bees exhibiting a rapid, full PER proceeded to the study.

Bees that were 7 days old were assessed for associative odor learning in a forward-paired conditioning paradigm. Clove oil (diluted 1:1000 in mineral oil; Sigma) was placed on filter paper in a 10 μL volume and inserted into a 0.5 mL glass syringe. The syringe was placed one inch from the bee, and connected to a solenoid-controlled air stream. The solenoid was powered by an Interval Generator 1830 (W.P. Instruments, Sarasota, FL, United States) to deliver a 5-s odor pulse (7 km/h). Vacuum suction was applied continuously. Three seconds into the pulse, a wooden applicator soaked with a 50% sugar solution was presented to the antennae. The bee was allowed to lick for 1 s. This sequence was repeated for three trials spaced 10 min apart. Three odor conditioning trials were found to be the least number of trials needed for long-term memory (Menzel et al., 2001). All experiments were performed between 9 AM and 1 PM under red light. These experiments took place over 4 days using 145 bees (30–39 each group).

Animals were tested for odor learning and memory of the conditioned odor 10 min after training. Bees were presented with a 5-s odor pulse and observed for proboscis extension immediately following the odor.

### Statistical Analysis

JMP 12.0 was used for all statistics. Amino acid concentrations were normalized using a log_10_ transformation and equal variances were found between groups. A three-factor ANOVA (pollen, infection, age) was applied to each amino acid individually, followed by a Tukey-HSD *post hoc* test. The effect of pollen on spore count was determined by transforming the data using log_10_ and applying a *t*-test. Odor learning and memory results were analyzed using non-parametric Wilcoxon/Kruskal–Wallis tests. To ascertain differences among treatment groups, *post hoc* Wilcoxon Each Pair tests were applied. Error bars denote standard error and significance was determined at α = 0.05 level.

## Results

### Pollen Modulates Amino Acid Levels in the Honey Bee Brain

**FIGURE 2:**
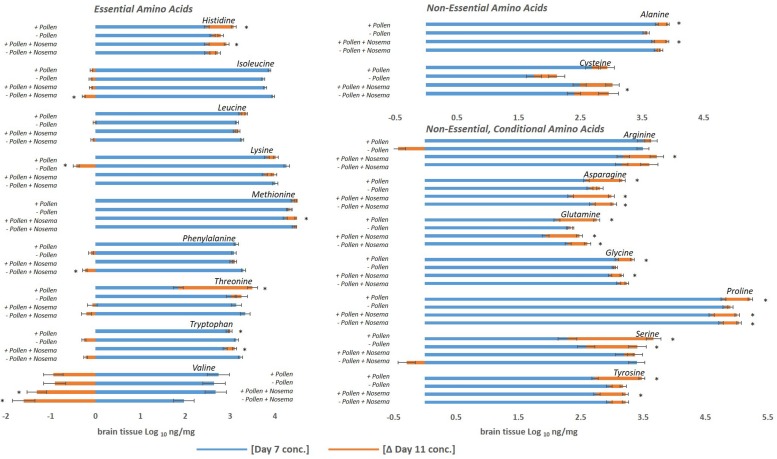
Amino acid levels in the honey bee brain with age: ± pollen, ± *Nosema ceranae*. The blue bars represent the mean concentration of amino acids present in bees at Day 7. The orange bars represent the mean difference in concentration in bees at Day 11 (Δ Day 11). In other words, blue bars represent the mean concentration at Day 7, and the orange bars denote the change with age by Day 11. Negative changes with age (Ex. Valine) are represented by orange bars to the left. They can be interpreted as the mean concentration difference from Day 7. For example, valine levels in pollen-fed bees was approximately 2.7 ng/mg and Day 11 was approximately 1.8 ng/mg. ^∗^Asterisks denote significant changes from Day 7 to Day 11 (*p* ≤ 0.05, three- way ANOVA, Tukey-HSD). Each group represents the average of 15 bees analyzed individually for 18 amino acids, *N* = 120.

The first question asked was how the honey bee brain responds to pollen. Age-matched pollen fed (+ P) bees were compared with pollen deprived (−P) bees at Day 7 and Day 11. At Day 7, concentrations of five amino acids were significantly different between + P and −P bees. These compounds included cysteine, isoleucine, lysine, threonine, and tryptophan (Figures 1, 2 and Tables 1, 2). Cysteine, a non-essential amino acid, fluctuated most with pollen. Cysteine was higher in the brains of + P bees compared to – P bees by 54% [*F*_(__7_,_89__)_ = 13.15, *p* ≤ 0.0001]. Isoleucine, an essential amino acid, was also significantly higher in + P bees, but with less variance (4%) [*F*_(__7_,_100__)_ = 11.15, *p* = 0.04]. Interestingly, isoleucine was the only essential amino acid found in higher concentration with pollen. Lysine, threonine, and tryptophan were lower with pollen by −10, −39, and −8%, respectively. For means and statistics, see Tables 1, 2: ‘Uninfected with Pollen Day 7.’

**TABLE 1:** Brain levels of amino acids in response to pollen, age and *Nosema ceranae* infection.

	Day 7			Day 11			*%* Change from Day *7 to Day 11*	
Amino acid	(+) Pollen	(−) Pollen	*Effect of Pollen?*	(+) Pollen	(−) Pollen	*Effect of pollen?*	(+) Pollen	(−) Pollen
**Uninfected Bees +/−*P***								
Alanine	3.72 ± 0.026	3.68 ± 0.027		3.9 ± 0.024	3.73 ± 0.028	***Higher	****5%	1%
Arginine	3.51 ± 0.100	3.5 ± 0.096		3.63 ± 0.100	3.07 ± 0.125	*Higher	3%	−12%
Asparagine	2.59 ± 0.046	2.65 ± 0.048		3.17 ± 0.044	2.81 ± 0.050	****Higher	****22%	6%
Cysteine	2.69 ± 0.105	1.75 ± 0.12	****Higher	2.94 ± 0.11	2.12 ± 0.13	****Higher	9%	21%
Glutamine	2.12 ± 0.049	2.32 ± 0.049		2.76 ± 0.048	2.34 ± 0.053	****Higher	****30%	1%
Glycine	3.08 ± 0.026	3.03 ± 0.027		3.33 ± 0.028	3.06 ± 0.031	****Higher	****8%	1%
Histidine	2.48 ± 0.054	2.61 ± 0.058		3.08 ± 0.054	2.79 ± 0.063	*Higher	****24%	7%
Isoleucine	3.88 ± 0.031	3.74 ± 0.033	*Higher	3.79 ± 0.033	3.63 ± 0.034	*Higher	−2%	−3%
Leucine	3.23 ± 0.032	3.15 ± 0.033		3.36 ± 0.032	3.13 ± 0.034	***Higher	4%	−1%
Lysine	3.82 ± 0.057	4.26 ± 0.062	****Lower	4.02 ± 0.057	3.83 ± 0.064		5%	*** −10%
Methionine	4.4 ± 0.044	4.31 ± 0.050		4.52 ± 0.044	4.33 ± 0.052		3%	0%
Phenylalanine	3.14 ± 0.050	3.09 ± 0.050		3.14 ± 0.048	2.99 ± 0.052		0%	−3%
Proline	4.79 ± 0.037	4.82 ± 0.038		5.22 ± 0.037	4.9 ± 0.041	****Higher	****8%	2%
Serine	2.29 ± 0.15	2.59 ± 0.14		3.67 ± 0.12	3.41 ± 0.14		****60%	**32%
Threonine	1.85 ± 0.11	3.03 ± 0.11	****Lower	3.5 ± 0.11	3.25 ± 0.14		****89%	7%
Tryptophan	2.73 ± 0.043	2.96 ± 0.046	*Lower	3 ± 0.043	2.88 ± 0.048		***10%	−3%
Tyrosine	2.95 ± 0.051	3.14 ± 0.047		3.48 ± 0.046	3.181 ± 0.051	**Higher	****18%	1%
Valine	2.74 ± 0.25	2.64 ± 0.25		1.8 ± 0.22	1.73 ± 0.25		−34%	−34%
***Nosema* bees +/− *P***								
Alanine	3.54 ± 0.026	3.74 ± 0.025	****Lower	3.77 ± 0.025	3.83 ± 0.025		****6%	2%
Arginine	3.18 ± 0.100	3.16 ± 0.100		3.72 ± 0.108	3.6 ± 0.141		*17%	14%
Asparagine	2.34 ± 0.046	2.69 ± 0.044	****Lower	2.99 ± 0.048	3.03 ± 0.046		****28%	****13%
Cysteine	2.49 ± 0.105	2.4 ± 0.105		3.02 ± 0.11	2.96 ± 0.16		*21%	23%
Glutamine	1.94 ± 0.053	2.3 ± 0.048	***Lower	2.48 ± 0.048	2.61 ± 0.049		***28%	***13%
Glycine	2.97 ± 0.027	3.11 ± 0.031	*Lower	3.16 ± 0.027	3.24 ± 0.028		***6%	4%
Histidine	2.48 ± 0.058	2.49 ± 0.058		2.92 ± 0.054	2.73 ± 0.056		****18%	10%
Isoleucine	3.78 ± 0.033	3.96 ± 0.031	**Lower	3.68 ± 0.033	3.7 ± 0.032		−3%	****−7%
Leucine	3.1 ± 0.032	3.27 ± 0.031	**Lower	3.19 ± 0.031	3.2 ± 0.032		3%	−2%
Lysine	3.77 ± 0.059	4.01 ± 0.059		3.98 ± 0.062	4.01 ± 0.059		6%	0%
Methionine	4.23 ± 0.046	4.43 ± 0.044	*Lower	4.49 ± 0.046	4.49 ± 0.050		**6%	1%
Phenylalanine	3.04 ± 0.048	3.3 ± 0.046	**Lower	3.1 ± 0.048	3.06 ± 0.050		2%	* −7%
Proline	4.6 ± 0.040	4.75 ± 0.037		5.01 ± 0.038	5.04 ± 0.038		****9%	**** 6%
Serine	3.2 ± 0.14	3.4 ± 0.13		3.37 ± 0.12	3.11 ± 0.14		5%	−9%
Threonine	3.14 ± 0.11	3.34 ± 0.11		3.07 ± 0.11	3.14 ± 0.11		−2%	−6%
Tryptophan	2.76 ± 0.044	2.96 ± 0.046	*Lower	3.1 ± 0.048	3.01 ± 0.044		****12%	2%
Tyrosine	2.89 ± 0.049	3.23 ± 0.046	****Lower	3.22 ± 0.046	3.22 ± 0.051		****11%	0%
Valine	2.68 ± 0.24	1.97 ± 0.23		1.37 ± 0.22	0.368 ± 0.25		**−49%	***−81%

Numbers indicate the average amino acid concentration (log_10_ ng/mg ± SEM). These values are reported for uninfected bees (top) and Nosema-infected bees (bottom). Day 7 and Day 11 refer to the age of the bee when brains were dissected, and values were separated according to whether bees were pollen-fed (+ Pollen) or unfed (− Pollen). “Effect of Pollen” denotes whether a pollen diet significantly affected an amino acid concentration. For example, “lower” indicates a drop in the average amino acid concentration with a pollen diet. The “Change from Day 7 to Day 11” indicates the percentage change from the Day 7 average amino acid concentration to the Day 11 average. Asterisks denote a significant change according to the following scale: * < 0.05, ** < 0.01, *** < 0.001, **** < 0.0001, three-way ANOVA, Tukey-HSD.

**TABLE 2:** Statistical values with pollen and age in bees ± infection.

				*Uninfected with Pollen*		*Infected with Pollen*		*Uninfected with Age*		*Infected with Age*	
Amino acids	df	*F*-value ANOVA	Prob > F	Day 7	Day 11	Day 7	Day 11	(+) Pollen	(−) Pollen	(+) Pollen	(−) Pollen
Alanine	7,98	17.75	< 0.0001*	0.97	0.0003*	< 0.0001*	0.57	< 0.0001*	0.92	< 0.0001*	0.14
Arginine	7,91	4.99	< 0.0001*	1	0.018*	1	0.99	0.99	0.15	0.011*	0.19
Asparagine	7,102	34.51	< 0.0001*	0.98	< 0.0001*	< 0.0001*	0.99	< 0.0001*	0.29	< 0.0001*	< 0.0001*
Cysteine	7,89	13.15	< 0.0001*	< 0.0001*	0.0003*	0.99	1	0.74	0.46	0.018*	0.1
Glutamine	7,103	26.74	< 0.0001*	0.109	< 0.0001*	< 0.0001*	0.56	< 0.0001*	1	< 0.0001*	0.0004*
Glycine	7,98	17.31	< 0.0001*	0.907	< 0.0001*	0.038*	0.52	< 0.0001*	0.99	0.0002*	0.052
Histidine	7,101	16.069	< 0.0001*	0.71	0.016*	1	0.22	< 0.0001*	0.49	< 0.0001*	0.068
Isoleucine	7,100	11.15	< 0.0001*	0.039*	0.0407*	0.0037*	0.99	0.41	0.38	0.32	< 0.0001*
Leucine	7,103	6.66	< 0.0001*	0.65	0.0001*	0.004*	1	0.098	1	0.53	0.71
Lysine	7,102	6.45	< 0.0001*	< 0.0001*	0.41	0.108	0.99	0.26	0.0002*	0.27	1
Methionine	7,100	4.79	0.0001*	0.87	0.11	0.046*	1	0.55	1	0.0024*	0.98
Phenylalanine	7,100	3.74	0.0012*	0.99	0.39	0.0047*	0.99	1	0.87	0.98	0.015*
Proline	7,104	25.71	< 0.0001*	0.99	< 0.0001*	0.16	0.99	< 0.0001*	0.79	< 0.0001*	< 0.0001*
Serine	7,93	10.25	< 0.0001*	0.85	0.85	0.97	0.86	< 0.0001*	0.0032*	0.98	0.81
Threonine	7,101	17.65	< 0.0001*	< 0.0001*	0.88	0.92	0.99	< 0.0001*	0.93	0.99	0.92
Tyrosine	7,100	14.36	< 0.0001*	0.13	0.0013*	< 0.0001*	1	< 0.0001*	0.99	< 0.0001*	1
Tryptophan	7,100	7.78	< 0.0001*	0.014*	0.56	0.045*	0.87	0.0008*	0.94	< 0.0001*	0.99
Valine	7,97	10.43	< 0.0001*	1	1	0.41	0.074	0.11	0.19	0.0034*	0.0003*

A three-way ANOVA with a Tukey Post hoc test was applied for each amino acid to determine how levels change in the brain with pollen and age in uninfected and infected bees. For example, “Uninfected with Pollen” denotes whether a pollen diet in uninfected bees altered an amino acid concentration and is separated according to Day 7 or Day 11 measurements. “Uninfected with Age” denotes whether an amino acid significantly varied from Day 7 to Day 11 and is separated according to pollen diet. *Asterisks highlight p < 0.05.

A different profile emerged in Day 11 bees (Table 1). Pollen elevated levels in the majority of amino acids tested, and non-essential amino acids in particular. These compounds included alanine, arginine, asparagine, cysteine, glutamine, glycine, proline, and tyrosine (Tables 1, 2). Cysteine levels fluctuated most with pollen (39%); while alanine (5%) varied least. Essential amino acids, histidine (10%), isoleucine (4%), and leucine (7%), were elevated with pollen, too. Other essential amino acids, however, maintained similar levels. Concentrations of lysine, methionine, phenylalanine, threonine, and valine did not change significantly with pollen, varying between 4 and 8%. For means and statistics, see Tables 1, 2: ‘Uninfected with Pollen Day 11.’

Pollen also influenced how amino acid concentrations changed with age (Figures 1, 2 and Tables 1, 2). Ten amino acids were higher in concentration from Day 7 to Day 11 (denoted as Δ%). The majority of these amino acids were non-essential. Amino acids such as alanine, asparagine, glutamine, glycine, proline, serine, and tyrosine elevated in concentration from Day 7 to Day 11, increasing on average from Δ 5 to 60% (Table 1). The largest changes were in serine (60%) and glutamine levels (30%), while the smallest changes were in alanine (5%) and glycine levels (8%). Essential compounds, such as histidine (Δ 24%), threonine (Δ 89%), and tryptophan (Δ 10%), also followed this pattern. However, most essential amino acids maintained similar levels with age. Amino acids like isoleucine, leucine, lysine, methionine, and phenylalanine varied as little as Δ 0–5%. Valine, a notable exception, lowered with age by 34%. For means and statistics, see Tables 1, 2: ‘Uninfected with Age + Pollen.’

In contrast, – P bees showed little change in amino acid levels with age (Figures 1, 2 and Tables 1, 2). Serine, in fact was the only compound found higher (Δ 32%) [*F*_(__7_,_93__)_ = 10.25, *p* = 0.003]. Lysine, though elevated marginally in + P bees (Δ 5%), significantly decreased in – P bees (Δ−10%) [*F*_(__7_,_102__)_ = 6.45, *p* = 0.0002]. For means and statistics, see Tables 1, 2: uninfected with Age ‘-Pollen.’

### Pollen Elevates *Nosema ceranae* Spore Count

**FIGURE 3:**
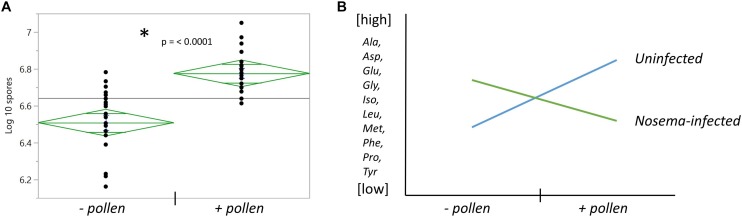
The effects of pollen and *Nosema ceranae* on spore count and amino acid relationships with pollen. **(A)** The number of spores counted in the abdomens of bees infected with *N. ceranae* ± pollen. *Asterisk denotes significance between groups measured by a *t*- test (*t*_(__38__)_ = 5.31, *p* ≤ 0.0001). **(B)** The general relationship between pollen and *N. ceranae* in 10 of 18 amino acids.

*Nosema ceranae* was determined to be the species of our *Nosema* inoculum based upon size and consistency with a *N. ceranae* positive control provided by the ARS-Beltsville laboratory. *Nosema apis* was not detected. The pollen-fed + *Nosema* bees (+P +N) had higher spore numbers than un-fed + *Nosema* bees (−P +N) (*t*_(__38__)_ = 5.31, *p* ≤ 0.0001) (Figure 3A). The uninfected bee abdomens in both the + P and −P groups contained zero spores (*N* = 40).

### *Nosema*-Infected Bees Have a Different Relationship With Amino Acid Levels and Pollen

We next asked whether similar relationships with pollen and age were found in *Nosema*-infected bees (+N). We compared +N bees that were pollen fed (+P +N) with those that were pollen-deprived (−P +N) at Day 7 and Day 11. Similar and different patterns emerged. At Day 7, pollen lowered levels in 10 of 18 amino acids in +N bees (Figures 1, 2 and Table 1) and these amino acids included both non-essential and essential compounds. The non-essential amino acids included alanine, asparagine, glutamine, glycine, and tyrosine that lowered with pollen between Δ−5 and Δ−16%. The essential amino acids also lowered with pollen between Δ−5 and Δ−8% and included isoleucine, leucine, methionine, phenylalanine, and tryptophan. By Day 11, however, pollen did not affect a single amino acid in +N bees. This result, at Day 11, was the largest difference with +N bees. Valine appeared to rise with pollen at Day 11, but did not yield a significant result [*F*_(__7_,_97__)_ = 10.43, *p* = 0.07]. For means and statistics, see Tables 1, 2: ‘Infected with Pollen.’

Pollen also influenced how amino acid concentrations changed with age in +N bees. When fed pollen, 11 amino acids were higher in concentration from Day 7 to Day 11. The majority of these amino acids were non-essential (Figure 2 and Tables 1, 2). These amino acids included alanine, arginine, asparagine, cysteine, glutamine, glycine, proline, and tyrosine. These amino acids rose with age between Δ 6 −28%. Asparagine and glutamine levels fluctuated most at 28%, while alanine and glycine fluctuated least at 6%. Three essential amino acids also followed this pattern including histidine (Δ 18%), methionine (Δ 6%), and tryptophan (Δ 12%). Valine was an exception. Valine lowered in concentration from Day 7 to Day 11 by 49%. For means and statistics, see Tables 1, 2: ‘Infected with Age + Pollen.’

Without pollen, amino acid levels remained mostly consistent or declined in −P +N bees (Figure 1 and Table 1). Only three amino acids were elevated from Day 7 to Day 11; and included asparagine (Δ 13%), glutamine (Δ 13%), and proline (Δ 6%). Isoleucine (Δ−7%), phenylalanine (Δ−7%), and valine (Δ−81%) were lower with age. For means and statistics, see Tables 1, 2: ‘Infected with Age – Pollen.’

### *Nosema* Infection and Pollen Show an Interactive Effect in the Brain

We then wondered whether pollen differentially affected amino acids in the brain with infection. We compared *Nosema*-infected bees (+N) with uninfected bees (−N) when age-matched and diet-matched (Table 3). We also examined the interaction between pollen and infection overall. Two main trends emerged: (1) With pollen, *Nosema* infection lowered levels of amino acids compared with −N bees; and (2) without pollen, *Nosema* infection raised levels of amino acids compared with −N bees. As a result, several amino acids showed an interaction effect between pollen and *Nosema* infection.

**TABLE 3:** Amino acids affected by *Nosema ceranae* infection by age and pollen in the honey bee brain.

(+) Pollen	Tukey-HSD	*Effect of infection*	(−) Pollen	Tukey-HSD	*Effect of infection*
**Day 7**					
Alanine	0.0001	****Lower	Cysteine	<0.0001	** Higher
Asparagine	0.0067	**Lower	Isoleucine	<0.0001	****Higher
Proline	0.017	*Lower	Phenylalanine	0.043	*Higher
Serine	0.0009	***Higher	Serine	0.0024	**Higher
Threonine	<0.0001	****Higher			
**Day 11**					
Alanine	0.0038	**Lower	Asparagine	0.031	*Higher
Glutamine	0.0023	**Lower	Cysteine	0.0043	**Higher
Glycine	0.0012	**Lower	Glutamine	0.0096	**Higher
Leucine	0.0057	**Lower	Glycine	0.0012	**Higher
Proline	0.0037	**Lower	Valine	0.0057	**Lower
Tyrosine	0.0041	**Lower			

Certain amino acids were found to be significantly affected by Nosema infection when brains were dissected at Day 7 and Day 11. These results changed based on whether a pollen diet was provided. “The Effect of Infection” denotes whether infected bees had a higher or lower amino acid concentration compared with uninfected bees. Asterisks denote a significant change * < 0.05, ** < 0.01, *** < 0.001, **** < 0.0001, three-way ANOVA, Tukey-HSD.

A significant interaction term between *Nosema* infection and pollen was found in 12 of 18 amino acids. In 10 of these compounds, a similar relationship was observed (Figure 3). When pollen was fed to +N bees, levels of many amino acids in the brain were lower. When pollen was deprived, levels of these same amino acids were higher. Amino acids that showed this pattern were a combination of non-essential and essential amino acids. The former included alanine (*Infection x Pollen*, *p* ≤ 0.0001), asparagine (*Infection x Pollen*, *p* ≤ 0.0001), glutamine (*Infection x Pollen*, *p* ≤ 0.0001), glycine (*Infection x Pollen*, *p* ≤ 0.0001), proline (*Infection x Pollen*, *p* ≤ 0.0001) and tyrosine (*Infection x Pollen*, p = 0.0014). The latter included isoleucine (*Infection x Pollen*, *p* ≤ 0.0001), leucine (*Infection x Pollen*, *p* ≤ 0.0001), methionine (*Infection x Pollen*, *p* = 0.0006), and phenylalanine (*Infection x Pollen*, *p* = 0.003). Cysteine and valine also showed an interaction term with infection and pollen, though slightly different. Cysteine levels rose in + P−N bees compared with – P −N bees; but in *Nosema*-infected bees, levels remained high regardless of diet (*Infection x Pollen*, *p* ≤ 0.0001). Valine showed the opposite. Levels rose in + P +N bees compared with −P +N bees, but was not affected by diet in uninfected bees (*Infection x Pollen*, *p* = 0.027).

### *Nosema*-Infected Bees With Lower Spore Numbers Differ From Nutritionally-Stressed Bees

We found that average spore numbers were higher in bees fed pollen (Figure 3). With this in mind, we wondered whether a low *N. ceranae* spore count (−P +N) differed from uninfected, nutritionally-stressed bees (−P −N). At Day 7, there were four amino acids that were different between −P +N bees and – P −N bees. These amino acids included both essential and non-essential amino acids: cysteine, isoleucine, phenylalanine, and serine. In all four compounds, −P +N bees had higher levels (Table 3). At Day 11, there were five amino acids different between −P +N bees and −P bees. Four of these amino acids were non-essential amino acids and included asparagine, cysteine, glutamine, and glycine. Again, in all four of these compounds, *Nosema*-infected bees had higher levels (Table 3). Interestingly, valine continued to be the exception in this comparison. Valine levels were lower in *Nosema*-infected bees.

### Does *Nosema* Infection Resemble Nutritional Stress?

We wondered whether amino acid levels could provide insight into whether *Nosema* infection is similar to nutritional stress. To evaluate whether *Nosema* infection in pollen-fed bees resembled pollen deprivation, we compared + P +N bees with −P −N bees at Day 11. We found that the addition of pollen in infected bees resulted in few changes compared with pollen-deprived bees. There were three of 18 amino acids that were different. These amino acids included arginine [*F*_(__7_,_91__)_ = 4.99, *p* = 0.0043], cysteine [*F*_(__7_,_89__)_ = 13.15, *p* ≤ 0.0001], and tryptophan [*F*_(__7_,_100__)_ = 7.78, *p* = 0.039]. In all three, levels were higher in + P +N bees.

### Odor Learning and Memory Is Affected by *Nosema* Infection and Pollen

**FIGURE 4:**
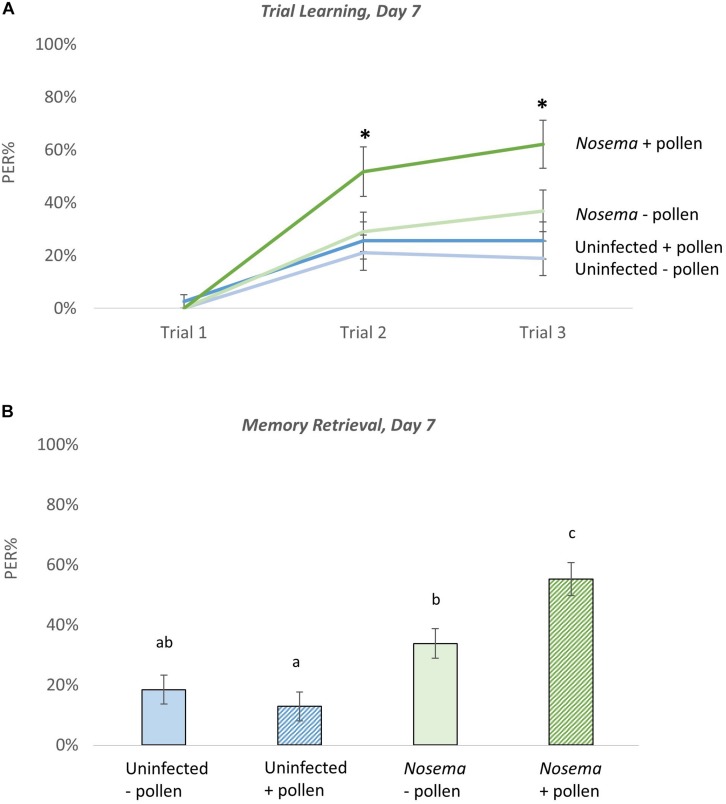
The effects of pollen and *Nosema ceranae* on odor learning and memory at Day 7. Proboscis extension response (PER) was measured among four groups of bees including: bees pollen (*N* = 39, 38) and *N. ceranae*-infected bees pollen (*N* = 30, 38). The PER response of each group was tested as bees were trained to associate an odor with a reward (Trial Learning, **A**). Memory retrieval **(B)** of the conditioned stimulus was measured 10 min after trial learning by presenting the odor alone. Significance(^∗^) was determined using separate Wilcoxon tests for Trial 2, Trial 3, and Memory Retrieval. Groups connected by the same letter are not significantly different.

The effects of *Nosema* infection and pollen in odor learning and memory behavior were analyzed in bees on Day 7 (Figure 4). During odor-associative training (when bees were conditioned to associate an odor with a sugar reward) there were differences found in PER performance (*N* = 145, 30–39 bees per group). After one associative odor pairing (Trial 2), the pollen-fed + *Nosema* bees (+P +N) exhibited better PER performance compared with −N bees; whether pollen-fed (+ P −N) [X^2^_(__3_, *_N_*_= 140__)_ = 2.18, *p* = 0.029], or pollen-deprived (−P −N) [X^2^_(__3_, *_N_*_= 140__)_ = 2.59, *p* = 0.0095]. PER performance was also elevated in +P +N bees compared with –P +N bees, though significance was borderline [X^2^_(__3_, *_N_*_= 140__)_ = 1.87, *p* = 0.061]. After the second odor pairing (Trial 3), effects of pollen and infection became more pronounced. The +P +N group exhibited better PER performance compared with uninfected bees, whether pollen-fed (+ P) [X^2^_(__3_, *_N_*_= 140__)_ = 2.98, *p* = 0.0028] or pollen-deprived (−P) [X^2^_(__3_, *_N_*_= 140__)_ = 3.55, *p* = 0.0004]. The +P +N bees also performed better than −P +N bees [X^2^_(__3_, *_N_*_= 140__)_ = 2.02, *p* = 0.042]. The PER performance between uninfected bees, in contrast, was not affected by pollen after one odor pairing (Trial 2: *Z* = 0.465, *p* = 0.64) or two odor pairings (Trial 3: *Z* = 0.69, *p* = 0.48).

When memory retrieval of the conditioned odor was tested, similar results of pollen and infection were observed [X^2^_(__3_, *_N_*_= 282__)_ = 34.47, *p* ≤ 0.0001)]. The +P +N group showed the highest recall of the conditioned odor, which was significantly higher than the other three groups: + P (*Z* = 5.26, *p* ≤ 0.0001), −P (*Z* = 4.42, *p* ≤ 0.0001), and −P +N (*Z* = 2.45, *p* = 0.014). The pollen-deprived + *Nosema* bees showed a slight, but significant increase in PER performance over the uninfected bees in both the pollen-deprived (*Z* = 2.13, *p* = 0.033) and the pollen-fed groups (*Z* = 3.055, *p* = 0.0022). The uninfected bees showed no difference in memory retrieval with pollen (*Z* = −0.95, *p* = 0.34).

## Discussion

### Amino Acids With Age, Pollen, and Behavior

**FIGURE 5:**
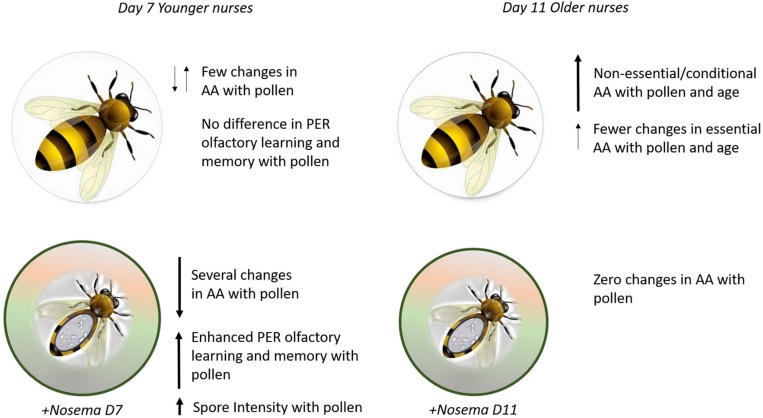
Summary figure. **(Top)** Bees fed pollen showed few changes in amino acids (AA) and no difference was found in olfactory learning and memory performance on Day 7. By Day 11, pollen-fed bees show higher levels of (AA) in the brain, especially in non-essential amino acids. **(Bottom)** *Nosema*-infected bees show a different profile with pollen. At Day 7, several AA in the brain are affected by pollen in addition to enhanced olfactory learning and memory behavior and spore intensity. By Day 11, pollen does not affect AA.

We gained insight into how the honey bee brain uses amino acids from pollen (Figure 5). Pollen is consumed primarily during the first 5 days of a honey bee’s life; which constitutes the majority of protein they will ever consume. Yet, the honey bee brain remains plastic and adaptive to behavioral change throughout adulthood. Our results showed that a pollen diet affects amino acid levels in the brain after this period of consumption. Pollen affected the brains of nurse-age bees at Day 7 and Day 11. Pollen also increased levels of amino acids with age. The majority of amino acids were present at a higher concentration at Day 11 compared with Day 7. In a previous study, this effect continued through Day 15 (Gage et al., 2018). These results suggest that pollen in early adulthood continues to affect neurodevelopment over time, which may affect later behaviors.

The majority of amino acids affected by pollen were non-essential. In other words, pollen enhanced amino acids that could be made by the body; while many essential amino acids required by diet maintained similar levels. De Groot in 1953 determined that ‘essential’ or ‘non-essential’ classification of amino acids in the honey bee followed closely with other animals (De Groot, 1953). He observed that many of the non-essential compounds were ‘stimulatory’ for honey bee growth. They were not essential for growth (measured in dry weight and nitrogen content in total body), but provided an enhancement, or ‘stimulatory effect’ when supplemented.

De Groot’s observation may also apply to the honey bee brain. Many of the non-essential amino acids affected by pollen (e.g., ala, arg, asp, cys, glu, gly, pro, tyr) have direct or indirect roles on the nervous system. These include effects on neurotransmission, functioning and development, or as ‘neuro-protectants’ against inflammation and oxidative stress (Wu, 2009). Cysteine, for example, was the amino acid whose concentration was most affected by pollen in our study. Cysteine is a limited resource in most insects, and necessary for glutathione production, an antioxidant found to neutralize reaction oxygen species and support immune function (McMillan et al., 2017). Tyrosine, another amino acid found to alter with pollen and infection is directly involved in protein phosphorylation, nitrosation, and sulfation; and is a precursor for dopamine: a neurotransmitter in the honey bee brain involved in behaviors like learning and memory (Agarwal et al., 2011).

The results we observed led us to consider how nutrients from pollen affect neural circuits. It is well-known that the honey bee brain is plastic with age, and behavior-regulating areas like the antennal lobe and the mushroom body appear to undergo continual optimization to support behaviors at a given time (Mercer, 2001). These changes are nutritionally expensive. We know from studies in humans and other mammals that pre-natal or early life nutrition can impact neural circuitry (Morgane et al., 2002; Nyaradi et al., 2013). Deficits in nutrition can impair processes like learning and memory that are dependent upon synapse formation and retraction. Our results suggest pollen consumption by honey bees may be similar. It may be that increasing levels of amino acids with age support the growth and adaptability of the honey bee brain. It needs to be addressed whether pollen leads to structural changes in areas like the antennal lobe, but our results indicate that early life nutrition in honey bees, similar to humans and other mammals, may have consequences for neurodevelopment, healthy aging and possibly behavior.

Olfactory learning and memory is an example of a complex neurobiological task that can be measured in the laboratory to assess the role of diet and behavior. We did not see an effect of pollen feeding on odor learning and memory in Day 7 bees. These bees performed poorly, and there was no difference found either in the association of an odor with a reward, or the recall. This may be because only a handful of amino acids were affected by pollen at Day 7 (cys, iso, lys, thr, trp), or this age may not support optimal odor learning and memory ability. Day 11 or a later age may have been a better choice. We chose Day 7 prior to our chemical analyses reasoning that the shortened lifespan of pollen-deprived bees (especially those that were infected) could be problematic to assess behavior at Day 11. There are reports, however, demonstrating that diet does influence olfactory learning and memory. Arien et al. (2015), found that fatty acids found in pollen, namely omega-3, were necessary for learning and memory performance in foragers. The role of amino acids in olfactory learning and memory remains to be tested.

### Essential Amino Acids

A surprise in this study was the consistency and low variance of many essential amino acids in the brain of bees fed pollen. Essential amino acids such as isoleucine, leucine, lysine, methionine, and phenylalanine, clustered tightly in our data set and varied with age between 0 and 5%. Only isoleucine and leucine were significantly higher with pollen on Day 7 or Day 11. Isoleucine, methionine and phenylalanine synthesize other amino acids found to vary significantly with pollen such as glutamine, alanine, cysteine, and tyrosine (Wu, 2009). Lysine is directly involved in nitric oxide synthesis, a known neurotransmitter to affect memory in bees and moths (Muller, 1996; Gage et al., 2013; Gage and Nighorn, 2014); and leucine regulates protein turnover through cellular mTOR signaling and gene expression (Wu, 2009). Given these roles, the low variation in essential amino acids may not indicate a lack of effect. It could mean these compounds exert effects within a narrow concentration range. For example, lysine levels marginally increased with age in bees fed pollen, while lysine levels significantly dropped in pollen-deprived bees (Figure 2). This finding suggests that a marginal increase in essential amino acids may be sufficient. These results can generate hypotheses for future studies on how essential amino acids might be used in adult brain development and behavior.

### Amino Acids Under Nutritional Stress

One of the most nutrient-sensitive tasks is nursing, which entails the ability to deliver brood food to developing larvae. The hypopharyngeal glands, which deliver this food, reduce in size and function under nutritional stress (Wang and Moeller, 1969, 1971; Alaux et al., 2011; Corby-Harris et al., 2016) and irregular nurse behavior develops (Schmickl and Crailsheim, 2001, 2002). Starved nurses will make fewer trips to the brood area (Schmickl and Crailsheim, 2002), and may even develop into precocious foragers (Schulz et al., 1998; Toth and Robinson, 2005).

We found that pollen deprivation changed the amino acid profile in the nurse brain, which may underpin irregular worker task behaviors of nurse bees observed with nutritional stress. While pollen-fed bees experience elevated levels of amino acids with age, levels remained consistent in pollen-deprived bees. Tryptophan may be an important amino acid to examine further. Tryptophan is required for hypopharyngeal gland growth (Fengkui et al., 2015), and we saw tryptophan in the brain rise with age in pollen-fed bees. In pollen-deprived bees, however, tryptophan in the brain remained consistent. Tryptophan is the precursor for serotonin, a neuromodulator and hormone that elevates in the brain with age (Schulz and Robinson, 1999) and affects nutrient intake, digestion, and feeding behavior in bees (French et al., 2014). Tryptophan may be useful as a supplement (Fengkui et al., 2015). It would be insightful to connect individual amino acids with hormonal signaling in the brain to understand the connection between hypopharyngeal gland physiology and irregular nurse behavior.

### Effects of Parasitic Stress From *Nosema ceranae*

Clues were gained as to how pollen affects the brain in *Nosema*-infected individuals. We saw that pollen feeding affected amino acids levels at Day 7, and that the levels were lower in infected bees fed a pollen diet. A prior study yielded similar results (Gage et al., 2018). However, this lowering of amino acid levels was found only with a pollen diet, rather than from infection alone. Pollen-deprived bees with *N. ceranae* showed significantly higher levels of amino acids. In fact, two thirds of the amino acids tested showed an interaction effect with pollen and infection.

Evidence from RNA-seq data indicates a link between diet and *N. ceranae* infection. Infection in nurses affected genes involved in amino acid production in the fat body (DeGrandi-Hoffman et al., 2018). The infection appeared to affect the tricarboxylic acid cycle (TCA cycle: ame00020), which generates energy from proteins, fats, and carbohydrates. This effect appears to be *N. ceranae* specific, as pollen deprivation alone does not prompt changes in TCA genes (Corby-Harris et al., 2014). Specifically, genes involved in the synthesis of valine, leucine, and isoleucine were highlighted (DeGrandi-Hoffman et al., 2018); which were amino acids also affected in this study. The DeGrandi-Hoffman study also found that the expression levels of 399 genes were differentially affected by *N. ceranae* and pollen type. These genes were involved in the TCA cycle, pyruvate metabolism (ame00620) and metabolic pathways (ame01100); which encompass numerous amino acid processes. Other studies have also pointed toward a connection between protein metabolism and *Nosema* sp. (Chen et al., 2013; Holt et al., 2013; Vidau et al., 2014; Glavinic et al., 2017; Li et al., 2019). In addition, *Nosema* has also been shown to affect gene expression in the brain (McDonnell et al., 2013; Doublet et al., 2016). The number of differentially expressed genes was small in response to infection (57 and 13; McDonnell et al., 2013; Doublet et al., 2016), with no significant functional groups identified, but genes affected included enzymes related to immune and antioxidant activity.

One may also consider the parasitic-nature of *N. ceranae* (Vidau et al., 2014; Mayack et al., 2015; Gage et al., 2018). There are host-parasite examples where parasites use nutrients from hosts to fuel replication (Zuzarte-Luís and Mota, 2018). For example, *Plasmodium* blood-stage parasites can sense the levels of host nutrient intake and modulate replication and virulence based on nutrient availability (Mancio-Silva et al., 2017). We found that a pollen diet significantly increased the number of *N. ceranae* spores within the honey bee midgut similar to other studies (Zheng et al., 2014; Fleming et al., 2015; Jack et al., 2016). This suggests a similar host-parasite dynamic may exist between *A. mellifera* and *N. ceranae*. If we contemplate a dispersal strategy for *N. ceranae*, it may be beneficial for the parasite to replicate inside a healthy host, who can forage and drift; and reduce replication in a starved host with a shorter lifespan.

We saw curious effects of infection on amino acids in the brain that may support this host-parasite dynamic. These results were most clear when comparing unfed bees. We found that *Nosema*-infected bees, even those with a lower spore count, differed from nutritionally-stressed bees. Eight amino acids were different between these groups, and all but one (valine) were present at higher concentration in infected bees (Table 2). One might guess that parasitic and nutritional stress in combination would result in the lowest levels of brain amino acids, but we did not find this to be the case. This combination of stressors appeared to boost amino acid levels in the brain, which may support the idea of ‘nutrient-sensing’ by *N. ceranae*. In addition, pollen-fed infected bees more closely resembled nutritionally-stressed bees. We compared groups at Day 11 (+P +N/− P −N) and found only three changes in amino acids: arginine, cysteine, and tryptophan, which were higher in infected bees. Still, infected bees live longer with pollen and protein content helps (Pasquale et al., 2013). These results may support the hypothesis that *N. ceranae* may sense nutrient levels in the host and modify its replication and virulence that weakens the host, but does not kill. Cysteine may be especially beneficial to infected bees. Our results showed generally higher levels of cysteine in *Nosema*-infected bees, and brain levels of cysteine varied most in all bees with pollen. Given cysteine’s role in glutathione production—a process known to boost immunity and neutralize reactive oxygen species— a cysteine supplement may be of benefit to fighting infection.

The interconnectedness of pollen and infection was demonstrated when testing olfactory learning and memory. Unlike uninfected bees, a pollen diet improved learning and memory performance. We saw 10 of 18 amino acids affected by pollen in infected bees, which may explain the learning and memory effect. Pollen-fed infected bees exhibited better responses during trial learning and in memory retrieval compared with both pollen-deprived infected bees and uninfected bees. It is unclear as to what this means, but there are a few possibilities. We saw this effect previously, in caged bees, and hypothesized that it could reflect the accelerated maturation (Gage et al., 2018), or precocious foraging, known to occur with infection (Goblirsch et al., 2013). Nurses tend to perform less well than foragers in the PER test (Ray and Ferneyhough, 1997), which may suggest heightened performance equates to accelerated maturation. In this paradigm, a pollen diet appears necessary for this effect. There are also examples of humans, rodents, and bumblebees displaying enhanced memory performance with stress (Schwabe et al., 2012; Muth et al., 2015). Circulating levels of stress hormones enhance memory-encoding processes (Schwabe et al., 2012), and this may be the case in infected bees fed pollen. Alternatively, better performance may reflect that infected bees are more motivated and sensitive to sugar. Infection increases sucrose sensitivity (Mayack and Naug, 2009) and this effect might improve performance in the PER tests. Infected bees generally showed better performance, but the unfed infected bees performed marginally better than uninfected bees. This result suggests sucrose sensitivity may not fully explain the enhanced learning and memory performance.

There are also limitations of this study to consider. The most notable is that these effects were observed in caged bees, without the social setting of the hive. This study also focused on amino acids from pollen, and it is likely that lipids, vitamins, sugars and minerals also contributed to spore proliferation and behavior. Moreover, we decided to mimic more natural *Nosema*-infection conditions by delivering spores to bees in infected gut contents. Purified spores were not used, and minute amounts of pollen grains may have been delivered along with micro-organisms found in gut contents. With these considerations, we propose that the honey bee brain is highly responsive to pollen, and a pollen diet continues to affect amino acids in the brain after consumption. Stress, either parasitic or nutritional changes this relationship. A better understanding of how pollen affects the brain of the honey bee provides insight into honey bee longevity, nutritional supplement improvement, and methods to understand and prevent the harmful effects of stress.

## Data Availability Statement

The datasets generated for this study are available on request to the corresponding author.

## Author Contributions

SG and GD-H conceived the study and wrote the manuscript. SG, NJ, SC, and MC performed the experiments and analyzed the data. All authors read and commented on the final version.

## Conflict of Interest

The authors declare that the research was conducted in the absence of any commercial or financial relationships that could be construed as a potential conflict of interest.
